# The endogenous peptide antisecretory factor promotes tonic GABAergic signaling in CA1 stratum radiatum interneurons

**DOI:** 10.3389/fncel.2014.00013

**Published:** 2014-01-28

**Authors:** Joakim Strandberg, Catarina Lindquist, Stefan Lange, Fredrik Asztely, Eric Hanse

**Affiliations:** ^1^Institute of Neuroscience and Physiology, Sahlgrenska Academy, University of GothenburgGöteborg, Sweden; ^2^Institute of Biomedicine, Sahlgrenska Academy, University of GothenburgGöteborg, Sweden

**Keywords:** tonic inhibition, GABA, IPSC, hippocampus, CA1

## Abstract

Tonic GABAergic inhibition regulates neuronal excitability and has been implicated to be involved in both neurological and psychiatric diseases. We have previously shown that the endogenous peptide antisecretory factor (AF) decreases phasic GABAergic inhibition onto pyramidal CA1 neurons. In the present study, using whole-cell patch-clamp recordings, we investigated the mechanisms behind this disinhibition of CA1 pyramidal neurons by AF. We found that application of AF to acute rat hippocampal slices resulted in a reduction of the frequency, but not of the amplitude, of spontaneous inhibitory postsynaptic currents (sIPSCs) in CA1 pyramidal neurons. Miniature inhibitory postsynaptic currents (mIPSCs), recorded in the presence of tetrodotoxin (TTX), were however not affected by AF, neither in CA1 pyramidal cells, nor in stratum radiatum interneurons. Instead, AF caused an increase of the tonic GABA_A_ current in stratum radiatum interneurons, leaving the tonic GABAergic transmission in CA1 pyramidal cells unaffected. These results show that the endogenous peptide AF enhances tonic, but not phasic, GABAergic signaling in CA1 stratum radiatum interneurons, without affecting tonic GABAergic signaling in CA1 pyramidal neurons. We suggest that this increased tonic GABAergic signaling in GABAergic interneurons could be a mechanism for the AF-mediated disinhibition of pyramidal neurons.

## Introduction

Antisecretory factor (AF) is an endogenous 43 kDa protein that is expressed in all mammalian tissues and plasma investigated so far (Johansson et al., 1995, 2009; Lange et al., 1999; Lange and Lönnroth, 2001; Ulgheri et al., 2010). The protein was named AF due to its capacity to counteract enterotoxin-induced intestinal hypersecretion, but later studies have shown that AF is also a potent anti-inflammatory agent (Johansson et al., 1997a, 2013; Eriksson et al., 2003; Davidson and Hickey, 2004a,b; Graber et al., 2011; Mane et al., 2011). Protein AF, which is also named S5a or RPn10, is a component of the 26S proteasome (Ulgheri et al., 2010). It can either be associated to the proteasome, or appear in a free form. This free form appears to have functions not associated with the proteasome.

The “active site” of AF, i.e., the part of the AF protein which effectuates the anti-secretory and anti-inflammatory action, can be isolated to an eight amino acid long sequence at the N-terminal part of the protein, IVCHSKTR (Johansson et al., 1997b). A somewhat larger peptide, AF-16, also including the active sequence, is more stable and therefore acceptable for experimental work (Jennische et al., 2008). AF has been demonstrated to counteract various forms of intestinal hypersecretion in clinical studies, and a number of experimental studies have demonstrated a protective, regulating effect of AF-16 in various models of tissue injury. Thus, AF-16 normalized the intracranial pressure and abolished mortality in rats with experimental herpes simplex 1 encephalitis (Jennische et al., 2008), while a high endogenous level of plasma AF prevented a rise in intracranial pressure and improved the cognitive functions in rats exposed to focal brain injury (Säljö et al., 2010). In a mouse colitis model treatment with AF decreased the extent of inflammation even in a late phase of the disease (Mane et al., 2011). Furthermore, the endogenous level of AF varies between mice strains. Thus, strains with a high AF mRNA expression are more resistant to autoimmune encephalitis than strains with a lower AF mRNA expression (Davidson and Hickey, 2004a). Taken together, the results of these studies suggest that the major effect of AF is to regulate/modulate hypersecretion and inflammatory reactions. The cellular mechanisms that mediate these anti-inflammatory and anti-secretory effects by AF are, however, largely unknown.

We have previously shown *in vitro* that AF-16 affects neuronal signaling in the brain (Kim et al., 2005). Specifically, application of AF-16 to acute rat hippocampal slices reduces GABAergic transmission onto CA1 pyramidal cells without affecting glutamatergic transmission. This disinhibitory effect was mimicked by feeding the rats with a diet containing hydrothermally processed cereals and by per oral administration of cholera toxin. In the present study we have investigated mechanisms behind this suppression of GABAergic transmission. We found that AF-16 enhances the tonic GABAergic current in CA1 stratum radiatum interneurons, without affecting the tonic GABAergic current in CA1 pyramidal neurons. We propose that the increased tonic GABAergic signaling in the GABAergic interneurons contributes to the AF-induced reduction of the feed-forward inhibition in this hippocampal region.

## Materials and Methods

### Hippocampal slice preparation

Hippocampal transverse slices (300–400 μm thick) were prepared from male P38–P60 Wistar rats, however in the experiments illustrated in Figure 6 both Wistar and Sprauge-Dawley rats were used. There was no difference in the effect of AF-16 between the two strains (see Section Results). Rats were anesthetized with isoflurane prior to decapitation using a guillotine. After decapitation the skull was opened and the brain hemispheres were put in a 4^˚^C solution containing (in mM): 110 cholineCl, 2.5 KCl, 1.25 NaH_2_PO_4_, 25 NaHCO_3_, 0.5 CaCl_2_, 7 MgCl_2_, 1.3 ascorbic acid and 7 D-glucose. Hippocampal slices were prepared using a vibratome (Microtome HM 650 V, Thermo Fisher Scientific, Loughborough, UK) in the same solution as above and then transferred to a storage chamber with a 25^˚^C artificial cerebrospinal fluid (ACSF) containing (in mM): 1.25 NaH_2_PO_4_, 124 NaCl, 26 NaHCO_3_, 3 KCl, 4 MgCl_2_, 2 CaCl_2_, 5 ascorbic acid, 4 D,L-lactic acid, 3 myo-inositol and 10 D-glucose. For experiments where slices were incubated in AF-16 the storage solution also contained 0.5 μg/ml AF-16 (this concentration of AF-16 was used in all experiments of this study). The slices were kept in the storage solution for 1–6 h before being transferred to the recording chamber. In the recording chamber the slices were perfused (2–3 ml/min) with ACSF containing (in mM): 1.25 NaH_2_PO_4_, 124 NaCl, 26 NaHCO_3_, 3 KCl, 2 MgCl_2_, 2 CaCl_2_ and 10 D-glucose. The perfusion ACSF also contained AF-16 in experiments where slices had been incubated in AF-16. All solutions were continuously bubbled with gas containing 95% O_2_ and 5% CO_2_ (pH 7.4) (AGA Gas AB, Lidingö, Sweden).

### Recording and analysis

CA1 pyramidal cells and stratum radiatum interneurons were visually identified using infrared differential interference contrast video microscopy mounted on an Olympus BX51WI microscope. Whole-cell patch-clamp recordings were performed with a patch clamp amplifier (EPC-9, Heka Elektronik, Lambrecht, Germany) at a sampling frequency of 10 kHz and filtered at 2.9 kHz. To record GABA_A_ receptor-mediated responses the neurons were voltage clamped at 0 mV and the pipette solution contained (in mM): 130 Cs-methanesulfonate, 2 NaCl, 10 HEPES, 0.6 EGTA, 5 Qx-314, 4 Mg-ATP and 0.4 GTP (pH 7.3, adjusted with D-gluconic acid). Patch pipettes (borosilicate, OD 1.5 mm, ID 0.86 mm) were pulled with a horizontal Flaming/Brown micropipette puller (P-97, Sutter Instrument Company, Novato, CA, USA) and they had a resistance of 2.8–6.6 MΩ. Series resistance was monitored using a 5 or 10 mV hyperpolarizing pulse and was not allowed to change more than 20%. All recordings were performed at room temperature. Analysis of miniature inhibitory postsynpatic currents (mIPSCs) and spontaneous IPSCs (sIPSCs) was performed using the Mini Analysis Program (version 5.6.28, Synaptosoft Inc, Fort Lee, NJ, USA). The first 100 miniature/spontaneous events at every given time point were analyzed. The cumulative plots shown in the figures were made from averaging the individual cumulative plots based on the normalized values from the time points indicated. Input resistance and holding current were measured using custom made software in Igor Pro (WaveMetrics Inc, Lake Oswego, OR, USA). The input resistance was measured using a hyperpolarizing pulse of 5 or 10 mV for 200–500 ms. Picrotoxin (PTX) (100 μM) was used to block GABA_A_ receptor mediated currents. The voltage-gated sodium channel blocker tetrodotoxin (TTX) (1 μM) was used to block action potentials when recording mIPSCs.

### Statistics

All data are expressed as means ± standard error of the mean. Statistical significance was evaluated using Student’s *t*-test for paired or independent data and Kolmogorov-Smirnov test for distributions.

### Drugs and chemicals

Chemicals were from Sigma-Aldrich (Steinheim, Germany) and Merck (Damstadt, Germany) except for TTX (Abcam Biochemicals, Cambridge, UK and Alomone labs, Jerusalem, Israel). The AF-16 peptide (VCHSK TRSNP ENNVG L) and the scrambled AF-16 peptide (GRSNK VENCL PHNST V) were synthesized with solid phase synthesis (Ross-Petersen AS, Copenhagen, Denmark).

## Results

### Antisecretory factor (AF-16) reduces the frequency of spontaneous inhibitory postsynaptic currents (IPSCs) onto CA1 pyramidal neurons

**Figure 1:**
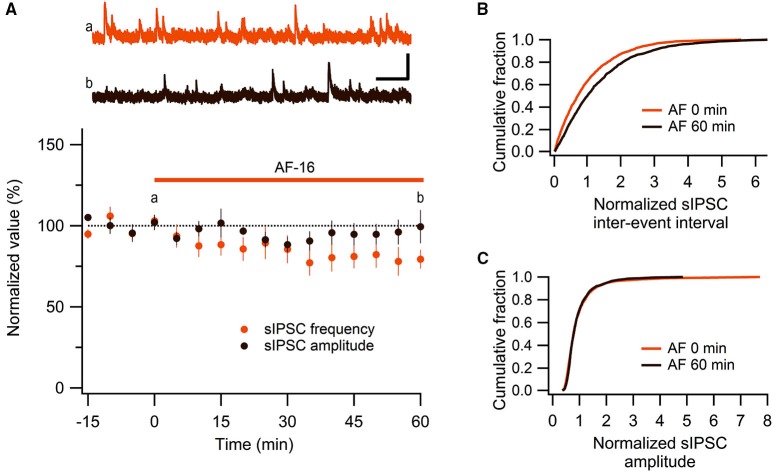
**AF-16 reduces the frequency of sIPSCs in CA1 pyramidal neurons. (A)** Summary graph of average (± s.e.m.) frequencies and amplitudes of recorded sIPSCs in CA1 pyramidal neurons (*n* = 8) during application of AF-16. Representative traces from time points a (orange trace before the application of AF) and b (dark brown trace 60 min after the application of AF) are shown at the top, scale bar represents 40 pA and 1 s. **(B)** Effect of AF-16 on the cumulative distribution of sIPSC inter-event intervals (*n* = 8). **(C)** Effect of AF-16 on the cumulative distribution of sIPSC amplitudes (*n* = 8).

**Figure 2:**
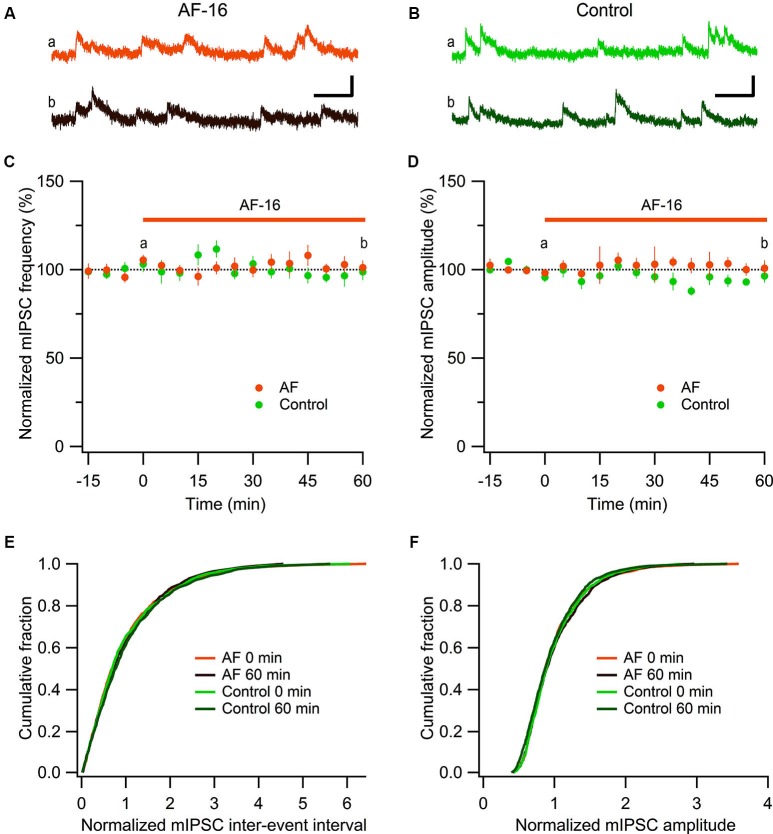
**No effect of AF-16 on mIPSCs in CA1 pyramidal neurons. (A)** Representative traces from time points a (orange trace before the application of AF) and b (dark brown trace 60 min after the application of AF) in **(C)** and **(D)** from an experiment with application of AF-16, scale bar represents 20 pA and 100 ms. **(B)** Representative traces from time points a (green trace at time “0 min”) and b (dark green at time “60 min”) in **(C)** and **(D)** from a control experiment without application of AF-16, scale bar represents 20 pA and 100 ms.** (C)** Summary graph of average (± s.e.m.) frequencies of recorded mIPSCs in CA1 pyramidal neurons exposed to AF-16 (*n* = 6) and controls not exposed to AF-16 (*n* = 7). **(D)** Summary graph of average (± s.e.m.) amplitudes of recorded mIPSCs in CA1 pyramidal neurons exposed to AF-16 (*n* = 6) and controls (*n* = 7). **(E)** Cumulative distributions of mIPSC inter-event intervals at 0 min and 60 min after AF-16 application (*n* = 6) and control (*n* = 7). **(F)** Cumulative distributions of mIPSC amplitudes at 0 min and 60 min after AF-16 application (*n* = 6) and control (*n* = 7).

We have previously shown, using extracellular field recordings, that AF-16 reduced evoked feed-forward GABAergic inhibition onto CA1 pyramidal neurons (Kim et al., 2005). In the present study, using whole-cell patch clamp recordings, we further examined the effect of AF-16 on GABAergic inhibition onto CA1 pyramidal neurons. We first investigated spontaneous action potential-dependent sIPSCs in CA1 pyramidal neurons. 50–60 min after applying AF-16 to the hippocampal slice there was a decrease in the frequency of sIPSCs to 78 ± 7% (*p* < 0.05, paired *t*-test) of the frequency obtained prior to the application, without a significant change in the averaged amplitude 95 ± 7% (*p* = 0.52, paired *t*-test) of the sIPSCs (*n* = 8) (Figure 1A). As described previously (Kim et al., 2005), the effect of AF-16 on GABAergic transmission developed slowly over more than 30 min. There was no run-down of phasic GABAergic signaling (in the presence of TTX) in control experiments (Figures 2C, D). In parallel with the average change in sIPSC frequency, the averaged cumulative distribution of sIPSC inter-event intervals before and 60 min after AF-application was significantly altered (Figure 1B) (*p* < 0.05, Kolmogorov-Smirnov test). The averaged cumulative distribution of sIPSC amplitudes was not significantly altered (Figure 1C) (*p* = 0.92, Kolmogorov-Smirnov test). This result confirms previous results (Kim et al., 2005) showing a disinhibitory effect of AF.

### Antisecretory factor (AF-16) does not alter miniature inhibitory postsynaptic current (mIPSC) amplitude or frequency onto CA1 pyramidal cells

To further investigate the reduced GABAergic inhibition onto CA1 pyramidal cells we examined the effect of AF-16 on spontaneous action potential-independent mIPSCs in CA1 pyramidal neurons. In the presence of 1 μM TTX, blocking all action potentials, we measured mIPSC frequency and amplitude before and 60 min after application of AF-16 (Figure 2). Application of AF-16 to the hippocampal slice had no significant effect on mIPSC frequency or amplitude (Figures 2A, B). The averaged frequency and amplitude of mIPCSs 60 min following application of AF-16 were 101 ± 4% and 101 ± 4%, respectively (*n* = 6), compared to before application of AF-16. To control for unspecific changes during whole-cell recordings we also measured mIPSC frequency and amplitude in control experiments without drug application (Figures 2B–D). The average frequency and amplitude of the mIPSCs were not significantly changed over 60 min in these experiments (99 ± 5% and 96 ± 4%, respectively, *n* = 7). The averaged cumulative distributions of inter-event intervals and amplitudes of mIPSCs before and 60 min after AF-application and corresponding controls were not significantly altered (Figures 2E, F) (AF: *p* = 0.99 for inter-event intervals and *p* = 0.98 for amplitudes, controls: *p* = 0.85 for inter-event intervals and *p* = 0.78 for amplitudes, Kolmogorov-Smirnov test). Taken together, AF-16 reduces the frequency of sIPSCs in CA1 pyramidal neurons without changing the frequency or amplitude of mIPSCs.

### Antisecretory factor (AF-16) enhances the tonic, but not phasic, GABA_A_ receptor-mediated current in CA1 stratum radiatum interneurons

**Figure 3:**
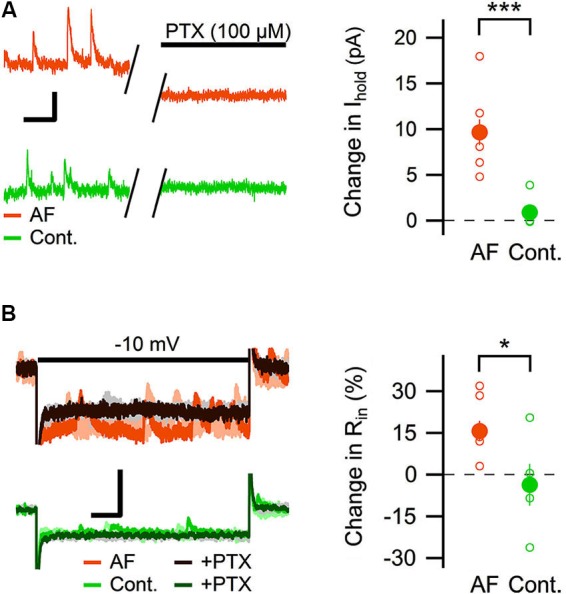
**AF-16 enhances tonic GABAergic transmission in CA1 stratum radiatum interneurons. (A)** Diagram showing the effect of PTX on the holding current in CA1 stratum radiatum interneurons incubated in AF-16 (*n* = 8) and control (*n* = 5). Open circles represent the individual experiments while the filled circles represent the average (± s.e.m.) values. On the left are representative traces before and after application of PTX, scale bar represents 10 pA and 250 ms. **(B)** Diagram showing the effect of PTX on the input resistance in CA1 stratum radiatum interneurons incubated in AF-16 (*n* = 8) and control (*n* = 5). Open circles represent the individual experiments while the filled circles represent the average (± s.e.m.) values. On the left are representative traces during a −10 mV test pulse before (orange/light orange and green/light green colors for AF and control, respectively) and after (dark brown/gray and dark green/gray colors for AF and control, respectively) application of PTX, scale bar represents 30 pA and 75 ms. * *p* < 0.05, *** *p* < 0.001.

**Figure 4:**
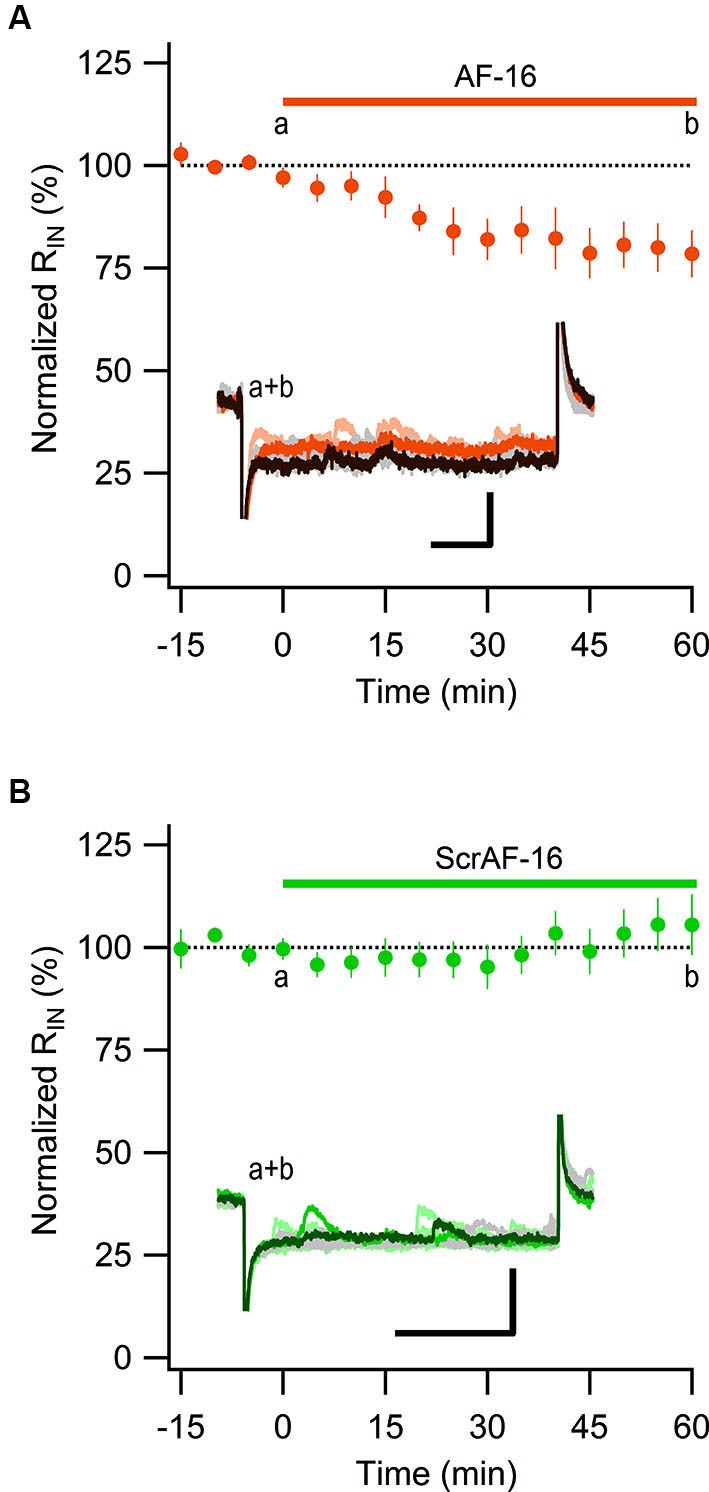
**AF-16 reduces the input resistance in CA1 stratum radiatum interneurons. (A)** Summary graph of average (± s.e.m.) input resistance during application of AF-16 in CA1 stratum radiatum interneurons (*n* = 8). Representative traces from time points a (orange/light orange colors) and b (dark brown/gray colors) are shown inside the graph, scale bar represents 30 pA and 75 ms.** (B)** Summary graph of average (± s.e.m.) input resistance during application of scrambled AF-16 in CA1 stratum radiatum interneurons (*n* = 8). Representative traces from time points a (green/light green colors) and b (dark green/gray colors) are shown inside the graph , scale bar represents 30 pA and 75 ms.

Most of the GABAergic inhibitory synaptic contacts onto CA1 pyramidal neurons are from local interneurons in the CA1 stratum radiatum (Klausberger, 2009; Bezaire and Soltesz, 2013). Since AF-16 decreased the frequency of action potential dependent sIPSCs, but not mIPSCs, onto CA1 pyramidal cells we speculated that the effect of AF-16 on GABAergic transmission could be due to a lowering of the intrinsic excitability of the interneurons making them less likely to signal onto the pyramidal cells. Increasing the tonic GABAergic current in the interneurons is one possible mechanism that could result in such a lowering of their intrinsic excitability. We therefore measured the tonic GABA_A_ receptor-mediated current in stratum radiatum interneurons by recording the change in holding current and input resistance after application of the GABA_A_ receptor channel blocker PTX in control slices and in slices that had been incubated in AF-16 for >1 h before measuring. Slices incubated in AF-16 had a larger tonic GABA_A_ receptor-mediated transmission both measured as the change in holding current (AF-16: 9.6 ± 1.4 pA, *n* = 8, compared to control: 0.9 ± 0.8 pA, *n* = 5, *p* < 0.001, unpaired *t*-test; Figure 3A) and measured as the change in input resistance (AF-16: 16 ± 4%, *n* = 8, compared to control: −4 ± 8%, *n* = 5, *p* < 0.05, unpaired *t*-test) (Figure 3B) after PTX application. A tonic GABA_A_ receptor-mediated transmission could be measured in all of the interneurons incubated in AF-16 (Figures 3A, B). As the effect of AF-16 on GABAergic transmission onto CA1 pyramidal cells develops slowly over more than 30 min, we also wanted to measure how the effect of AF-16 on tonic GABAergic transmission in CA1 stratum radiatum interneurons develops over time. We measured the effect of AF-16 as the change in input resistance over 60 min after application of AF-16 to the ACSF. 60 min after the application of AF-16 the input resistance had decreased by −22 ± 6% (*n* = 8) (Figure 4A), significantly different from the observed effect on the input resistance by a scrambled AF-16 peptide (6 ± 7%, *n* = 8, *p* < 0.05, unpaired *t*-test) (Figure 4B).

**Figure 5:**
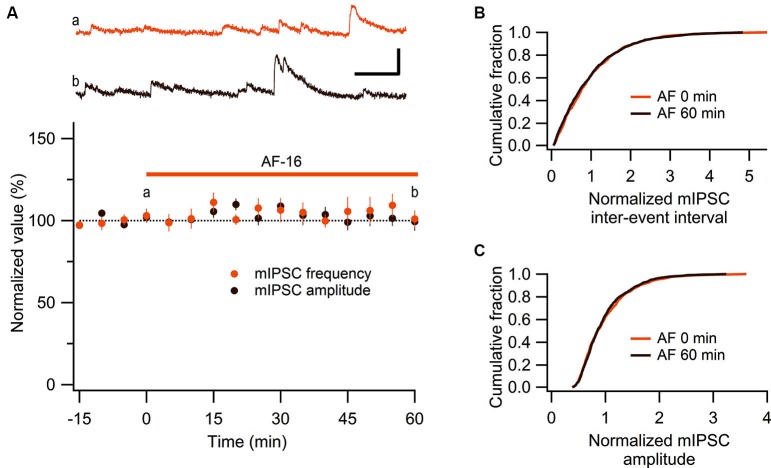
**No effect of AF-16 on mIPSCs in CA1 stratum radiatum interneurons. (A)** Summary graph of average (± s.e.m.) frequencies and amplitudes of recorded mIPSCs in CA1 stratum radiatum interneurons (*n* = 6) during application of AF-16. Representative traces from time points a (orange trace before the application of AF) and b (dark brown trace 60 min after the application of AF) are shown at the top, scale bar represents 20 pA and 100 ms. **(B)** Effect of AF-16 on the cumulative distribution of mIPSC inter-event intervals (*n* = 6). **(C)** Effect of AF-16 on the cumulative distribution of mIPSC amplitudes (*n* = 6).

These results show that AF causes an increased tonic GABAergic signaling onto GABAergic interneurons. We next wanted to examine whether phasic GABAergic inhibition onto these interneurons was affected by AF. We therefore recorded mIPSCs in stratum radiatum interneurons before and after the application of AF-16. As can be seen in Figure 5A there was no significant change in the frequency (105 ± 5%) or the amplitude (101 ± 5%) of mIPSCs 50–60 min following application of AF-16 (*n* = 6). The averaged cumulative distributions of inter-event intervals and amplitudes of mIPSCs before and 60 min after AF-application were not significantly altered (Figures 5B, C) (*p* = 0.99 and *p* = 0.99, respectively, Kolmogorov-Smirnov test). These results thus indicate that AF enhances tonic, but not phasic, GABAergic inhibition onto these interneurons.

### The tonic GABA_A_ receptor-mediated current in CA1 pyramidal cells is not altered by antisecretory factor (AF-16)

**Figure 6:**
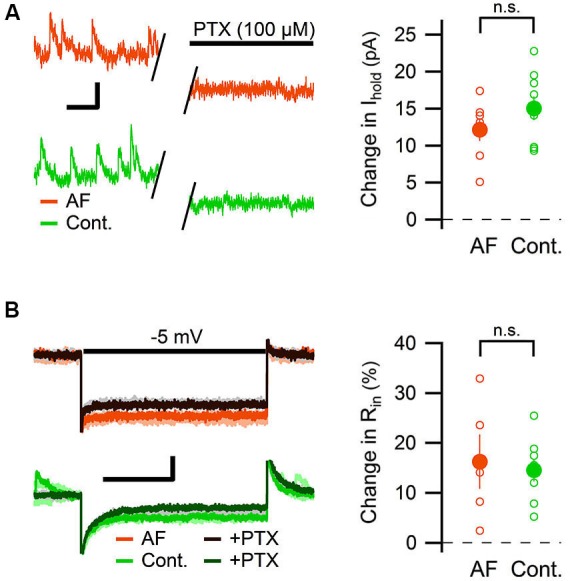
**AF-16 does not enhance tonic GABAergic transmission in CA1 pyramidal neurons. (A)** Diagram showing the effect of PTX on the holding current in CA1 pyramidal neurons incubated in AF-16 (*n* = 7) and control (*n* = 9). Open circles represent the individual experiments while the filled circles represent the average (± s.e.m.) values. On the left are representative traces before and after application of PTX, scale bar represents 10 pA and 250 ms. **(B)** Diagram showing the effect of PTX on the input resistance in CA1 pyramidal neurons incubated in AF-16 (*n* = 5) and control (*n* = 8). Open circles represent the individual experiments while the filled circles represent the average (± s.e.m.) values. On the left are representative traces during a −5 mV test pulse before (orange/light orange and green/light green colors for AF and control, respectively) and after (dark brown/gray and dark green/gray colors for AF and control, respectively) application of PTX, scale bar represents 30 pA and 75 ms. n.s. not significant (*p* > 0.05).

The change in tonic GABAergic transmission onto interneurons could depend on a change in the expression levels or kinetics of the GABA receptors, but it could also depend on an increased level of ambient GABA (Jensen et al., 2003; Glykys and Mody, 2007; Song et al., 2013). An increased level of ambient GABA is expected to also enhance the tonic GABAergic transmission in pyramidal neurons. We therefore measured the effect of AF-16 on the tonic GABA_A_ receptor-mediated current in CA1 pyramidal neurons. By using the same analytical approach used to measure the tonic GABA_A_ receptor-mediated tonic current in the interneurons, AF-16 did not significantly alter the change in holding current (AF-16: 12 ± 1.5 pA, *n* = 7, compared to control: 15 ± 1.6 pA, *n* = 9, *p* = 0.22, paired *t*-test) (Figure 6A) or in input resistance (AF-16: 16 ± 5%, *n* = 5, compared to control: 15 ± 2%, *n* = 8, *p* = 0.74, paired *t*-test) (Figure 6B) after PTX application. In this specific set of experiments both Wistar and Sprague-Dawley rats were used. The results were pooled since there was no significant difference between the two strains (changes in holding current after PTX applications were: 12.8 ± 1.8 pA, *n* = 4 in Wistar AF-16 treated compared to 11.2 ± 3.1 pA, *n* = 3 in Sprague Dawley AF-16 treated, *p* = 0.65 (independent samples *t*-test) and 14.1 ± 1.7 pA, *n* = 7 in Wistar control compared to 18.4 ± 4.3 pA, *n* = 2 in Sprague Dawley control, *p* = 0.29 (independent samples *t*-test)).

## Discussion

In the present study we have investigated how the endogenous peptide AF affects GABAergic transmission onto pyramidal neurons and stratum radiatum GABAergic interneurons in the hippocampus. In pyramidal neurons we found that AF causes a reduction in the frequency of action potential-dependent, but not of action potential-independent, spontaneous IPSCs, and that AF did not affect the tonic GABAergic transmission. In interneurons, on the other hand, we found that AF enhances the currents through tonically active GABA_A_ receptors. Phasic GABA_A_ receptor-mediated synaptic transmission onto the interneurons, as measured as mIPSCs, was not affected. We thus identify AF as positive neuromodulator of tonic GABAergic signaling specifically on GABAergic interneurons.

We propose that the increase of the tonically active GABA_A_ receptor-mediated current, induced by AF, results in a decreased excitability of the interneurons. It is however not obvious that an increased tonic GABA current results in decreased excitability since GABAergic interneurons, in contrast to pyramidal neurons, do not exhibit a developmental shift from a depolarizing to a hyperpolarizing reversal potential for GABA_A_ receptor-mediated currents (Banke and Mcbain, 2006). More specifically, stratum radiatum interneurons have been shown to exhibit a reversal potential for tonic GABA_A_ receptor-mediated currents (−62 mV) that is more depolarized than the resting membrane potential (−72 mV), but more hyperpolarized than the threshold for action potential (−44 mV) (Song et al., 2011). Song et al. (2011) further showed that a low increase of the GABA_A_ conductance can actually increase the excitability of these interneurons (via a depolarization), whereas a larger increase of the GABA_A_ conductance results in a decreased excitation (when the shunting currents overrides the depolarization). Our findings suggest that the AF-induced increase of the tonic GABA_A_ receptor-mediated current results in a decreased interneuronal excitability. Importantly, application of AF resulted in a reduced, not increased, frequency of spontaneous, action-potential dependent, IPSCs in CA1 pyramidal neurons. This result is also consistent with our previously reported reduction of evoked feed-forward inhibition in CA1 pyramidal neurons (Kim et al., 2005). Moreover, the temporal development of the enhanced tonic GABA_A_ receptor currents in the interneurons paralleled that of the decreased evoked (Kim et al., 2005), and spontaneous (Figure 1), inhibitory synaptic activity onto the CA1 pyramidal neurons.

In the present study we have not examined the mechanisms by which AF increases the tonic GABA_A_ receptor-mediated current in the stratum radiatum interneurons. We envision two main, non-exclusive, possible mechanisms: an increased concentration of ambient GABA, or more functionally active extrasynaptic GABA_A_ receptors. An increased concentration of ambient GABA, produced by, for example, inhibition of GABA transporters, seems unlikely since we did not find any enhancement of the tonic GABAergic current in CA1 pyramidal neurons. It can be noted that tonic GABAergic currents may be difficult to detect in CA1 pyramidal neurons because of prominent outward rectification (Pavlov et al., 2009). In this study we have, however, recorded GABAergic currents at 0 mV (using a low intracellular chloride concentration), and we observed prominent tonic GABAergic currents in the CA1 pyramidal neurons, but no enhancement by AF. We did not find any significant tonic GABAergic signaling in the GABAergic interneurons under baseline conditions. This discrepancy between our finding and that reported by others (Semyanov et al., 2003; Scimemi et al., 2005; Song et al., 2013) seems to be quantitative rather than qualitative. Indeed there are other reports of non-significant tonic GABAergic signaling in interneurons in the CA1 region (Bieda and Maciver, 2004), as well as in somatostatin-positive interneurons in layer 2/3 of the frontoparietal cortex (Vardya et al., 2008). It appears that this difference relates to differential basal conditions including different strains, gender and age of the animals used (Marchionni et al., 2007).

In CA1 pyramidal neurons tonic GABAergic signaling is mediated mainly via α5-containing GABA_A_ receptors, whereas δ-containing GABA_A_ receptors mediate tonic GABAergic signaling in dentate granule cells and many GABAergic interneurons (Gao and Fritschy, 1994; Sperk et al., 1997; Glykys et al., 2008; Mann and Mody, 2010; Ferando and Mody, 2012). A differential modulation of tonic GABAergic signaling mediated by different types of GABA_A_ receptors has previously been reported (Glykys et al., 2007; Jin et al., 2011; Tao et al., 2013). It is thus possible that the presently found differential effect of AF on tonic GABAergic signaling could be explained by a selective increase in the number and/or efficacy of extrasynaptic GABA_A_ receptors in GABAergic interneurons that normally express δ-containing GABA_A_ receptors. Such an increase of extrasynaptic GABA_A_ receptors could be mediated by exocytosis and membrane insertion of GABA_A_ receptors (Saliba et al., 2012). We note, however, that the AF-induced increase of tonic GABAergic signaling in the interneurons develops over tens of minutes, indicating that the putative exocytosis might include synthesis of new receptors. It is also important to note that the proposed AF-induced membrane insertion of new GABA receptors is exclusive for the extrasynaptic membrane since AF did not induce any increase of the mIPSC amplitude in the GABAergic interneurons, or in the CA1 pyramidal neurons. Our study thus adds to the concept of differential modulation of phasic and tonic GABAergic signaling. It also adds to the emerging concept of differential modulation of tonic GABAergic signaling in different types of neurons. Since the modulation by different neuromodulators likely differs with gender, age, species, strain, etc, the emerging concept of differential modulation may also help to explain the reported variation in tonic GABAergic signaling in different studies.

In the CA1 region the interneurons can be divided into different groups based on location, morphology, physiological and molecular features (Ascoli et al., 2008; Klausberger and Somogyi, 2008). The cell bodies of the interneurons in the CA1 region are located in stratum radiatum as well as stratum lacunosum-moleculare, pyramidale and oriens. In the present study we only investigated interneurons with their cell bodies located in the stratum radiatum, with no further characterization. This selection likely includes cholecystokinin-positive Schaffer-collateral associated and apical dendrite innervating interneurons, but excludes parvalbumin-positive interneurons (Klausberger and Somogyi, 2008). We therefore do not know if our findings also apply to interneurons located in other parts of the CA1 region. However, all the interneurons investigated displayed similar effects elicited by AF. Moreover, the about 20% reduction in the frequency of sIPSCs in CA1 pyramidal neurons by AF (Figure 1) is similar to the reduction of evoked feed-forward inhibition (Kim et al., 2005), suggesting that the presently described effect of AF may be general among hippocampal interneurons.

The modulation of interneuronal extrasynaptic GABA_A_ receptors by AF adds to the concept that extrasynaptic GABA_A_ receptors are an important target for neuromodulation (Garcia et al., 2010; Jin et al., 2011; Ferando and Mody, 2012; Tao et al., 2013). GABA_A_ receptors are however not only expressed on neurons in the central nervous system, but also in the enteric nervous system (Krantis, 2000), as well as on non-neuronal cells such as immune cells (Bjurstom et al., 2008) and endocrine pancreatic cells (Jin et al., 2013). Dysregulation of GABA_A_ receptors on these cells has been implicated in immunological disorders (Bjurstom et al., 2008) and diabetes (Taneera et al., 2012). The cellular mechanisms behind AF’s anti-inflammatory and antisecretory effects have not been established, but our present results suggest the possibility that one such cellular mechanism is mediated via a positive modulation of GABA_A_ receptors on certain neuronal and non-neuronal cells.

## Author contributions

Joakim Strandberg, Stefan Lange, Fredrik Asztely and Eric Hanse designed research; Joakim Strandberg and Catarina Lindquist performed research; Joakim Strandberg analyzed data; Joakim Strandberg, Fredrik Asztely and Eric Hanse wrote the paper. All authors read and commented on the final draft of the paper.

## Conflict of interest statement

The authors declare that the research was conducted in the absence of any commercial or financial relationships that could be construed as a potential conflict of interest.
